# Linking gender differences with gender equality: A systematic-narrative literature review of basic skills and personality

**DOI:** 10.3389/fpsyg.2023.1105234

**Published:** 2023-02-16

**Authors:** Marco Balducci

**Affiliations:** Department of Social Research, University of Turku, Turku, Finland

**Keywords:** gender equality paradox, gender equality, gender differences, basic skills, personality

## Abstract

There is controversy regarding whether gender differences are smaller or larger in societies that promote gender equality highlighting the need for an integrated analysis. This review examines literature correlating, on a national level, gender differences in basic skills—mathematics, science (including attitudes and anxiety), and reading—as well as personality, to gender equality indicators. The aim is to assess the cross-national pattern of these differences when linked to measures of gender equality and explore new explanatory variables that can shed light on this linkage. The review was based on quantitative research relating country-level measures of gender differences to gender equality composite indices and specific indicators. The findings show that the mathematics gender gap from the PISA and TIMMS assessments, is not linked to composite indices and specific indicators, but gender differences are larger in gender-equal countries for reading, mathematics attitudes, and personality (Big Five, HEXACO, Basic Human Values, and Vocational Interests). Research on science and overall scores (mathematics, science, and reading considered together) is inconclusive. It is proposed that the paradox in reading results from the interrelation between basic skills and the attempt to increase girls’ mathematics abilities both acting simultaneously while the paradox in mathematics attitudes might be explained by girls being less exposed to mathematics than boys. On the other hand, a more nuanced understanding of the gender equality paradox in personality is advanced, in which a gene–environment-cultural interplay accounts for the phenomenon. Challenges for future cross-national research are discussed.

## 1. Introduction

Despite Western countries having considerably advanced in gender equality, gender horizontal segregation remains among the main drivers of economic gender inequality (Cech, 2013). Women have entered the labor market at increasingly high rates since the 70s, nevertheless, they often still work in specific sectors with substantial effects on their income (Cortes and Pan, 2018). Gender segregation is already visible at the educational level where girls are overrepresented in disciplines such as Social Sciences and Humanities; these subjects are characterized by lower labor market prospects and income (van de Werfhorst, 2017). On the other hand, boys prefer STEM fields which offer high-salaried and more status-related careers (Barone and Assirelli, 2020). To explain the phenomenon, scholars in sociology and psychology have been particularly interested in basic skills and personality gender variances due to their influence on gendered career choices and outcomes (Rosenbloom et al., 2008; Dekhtyar et al., 2018; Stoet and Geary, 2018).

Regardless of doubts about their magnitude (Hyde, 2005; Archer, 2019; Hirnstein et al., 2022), gender differences in basic skills and personality are well-established in the literature (Halpern, 2000; Halpern et al., 2007; Geary, 2010; Weisberg et al., 2011). The gender gaps favoring boys in mathematics and science are close to zero on average but observable at the upper and lower tails of the distribution (Halpern et al., 2007; Wai et al., 2018). Conversely, differences in reading skills (women > men) are more pronounced and already noticeable when comparing men’s and women’s statistical means (Halpern, 2000; Moè et al., 2021). Regarding personality (Big Five, HEXACO, Basic Human Values, and Vocational Interests), gender variances, although small to medium, occur across models and share a similar pattern. On the one hand, women score higher in negative emotions and reciprocity as well as prefer to “work with people.” On the other hand, men have more realistic preferences and regard status-related values more (Schwartz and Rubel, 2005; Schmitt et al., 2008; Su et al., 2009; Lee and Ashton, 2018). On a national level, however, the link between these gender differences and gender equality, measured using conventional indicators such as the World Economic Forum’s Global Gender Gap Index (GGI), remains unclear with scholars making contrasting predictions.

Numerous social-role theories of gender differences expect that the gaps between men and women will decrease as equality between them is achieved (Eagly and Mitchell, 2004; Else-Quest et al., 2010). These theories argue that cognitive and personality gender differences are derived from socially constructed gender identities based on erroneous essential beliefs (stereotypes) that men and women are intrinsically different (Wood and Eagly, 2013). Gender stereotypes originate from the division of labor in ancient hunter-gatherer societies, in which greater strength allowed men to engage in more power-related activities, while women were tasked with nurturing duties because of their ability to breastfeed (Eagly and Wood, 1999). Stereotypes would emerge early in life, with elementary school children already consistently engaging in gender essentialism, gender stereotyping, and implicit gender associations (Meyer and Gelman, 2016). Parents, teachers, and friends are responsible for reinforcing them, rewarding children for behaving according to gendered expectations (Gunderson et al., 2012), thereby making gender a “primary framing device for social relations” (Ridgeway, 2006). As a result, boys and girls grow up into adults who have gender-specific roles in society and experience gender-conforming environments that shape their distinct skills and personalities (Diekman and Schneider, 2010). The common assumption underlying these theories predicts that essentialist beliefs decrease in countries with higher gender equality. If this is true, empirical research will find smaller gender differences in more gender-equal nations.

Other studies have theorized an opposite trend, with men and women becoming increasingly dissimilar in gender-equal countries (Charles and Bradley, 2009; Kaiser, 2019). Recently, Stoet and Geary (2018) labeled this phenomenon “the gender equality paradox.” Some have proposed that this paradox results from an emphasis on individualism and a societal system designed to accommodate women in what is perceived to be their gendered role (Charles and Grusky, 2018). Others have applied an evolutionary approach and argued that in less unequal environments, men and women freely express their intrinsic differences as the privileged access to resources in “more prosperous and more egalitarian” societies favors the emergence of specific gender-evolved behaviors (Schmitt et al., 2008).

Although the topic of gender difference has been widely discussed, whether men and women become progressively similar or different when greater equality between them has been achieved remains uncertain. This paper reviews several theories hypothesizing contrasting patterns, and then turns to the recent scientific debate on gender differences in basic skills from the PISA and TIMMS assessments, as well as personality (Big Five, HEXACO, Basic Human Values, and Vocational Interests) to consider how they relate to measures of gender equality on a national level. Several challenges for future cross-national research are also highlighted. Specifically, the present review indicates that the correlation between gender differences in mathematics and gender equality may derive from the lack of country-level effects in the models, while ecological stress (food consumption and historical levels of pathogen prevalence) may confound the results for personality. In addition, the paper examines explanations of the paradox in different domains and proposes a novel theory to explain the gender equality paradox in personality, where a “feedback-loop” effect (gene–environment-culture interplay) might account for the phenomenon.

## 2. Methods

The narrative approach was assessed to be the most suitable method for this study. Compared to more analytical methods, it allows for deeper insights into the ongoing debate (Graham, 1995). However, issues may arise with this method due to bias in paper selection and interpretation (Dijkers, 2009). To avoid these issues, the author implemented a systematic approach based on PRISMA guidelines together with the narrative method.

### 2.1. Eligibility criteria

To be eligible for inclusion, papers had to have been published between 2009 and 2022, and they had to describe quantitative cross-national research analyzing gender differences associated with measures of gender equality (composite indices or specific indicators) utilizing international data. The selected studies were divided into two groups—basic skills and personality—then further divided into multiple subgroups: mathematics, science (including attitudes and anxiety), reading, and overall scores for basic skills, as well as the Big Five, the HEXACO model, basic human values and vocational interests for personality factors. Since they had fewer available papers, the Big Five and HEXACO, as well as basic human values and vocational interests categories were combined.

### 2.2. Information sources

Published studies were selected from Scopus, Web of Science, Social Science Database, and Google Scholar. The final search was conducted on all databases in November 2022.

### 2.3. Search strategy

The research focused on gender differences in basic skills and personality due to their strong relationships with horizontal gender segregation. Thus, the main search words were “gender/sex differences in mathematics/reading/science,” “gender/sex differences in personality,” “gender/sex differences in basic human values” and “gender/sex differences in vocational interests.” The search was then refined using “gender equality/egalitarianism/inequality” as parameters.

### 2.4. Selection process

Only papers published in English were considered, and they were selected based on their titles, abstracts, and keywords. This study’s author was primarily responsible for the selection, although two other scholars supervised the process and ensured systematic application of the selection criteria.

### 2.5. Items

Ninety-one papers were preselected; 35 were excluded after deeper screening because they did not match the selection criteria. An additional 25 studies were excluded because they studied gender differences outside the domains of interest. Consequently, 31 papers were included in the study.

## 3. Overview of gender differences in basic skills and personality and their possible relation to gender equality

### 3.1. Gender differences

On a national level, gender differences in basic skills and personality have been repeatedly described. Research has shown that boys slightly outperform girls in complex mathematical riddles (Reilly et al., 2019); this difference has been associated with men’s overrepresentation in STEM fields (Dekhtyar et al., 2018). Although the difference approaches zero, gaps are especially visible among the top and lower performers because of the higher variability in boys (Lindberg et al., 2010; Wai et al., 2018). Stated otherwise, while there are barely any differences on average, the men’s distribution has a flatter curve, yielding higher values at both the lowest and highest ends. Similarly, men appear to have a small advantage over women in science, with differences particularly visible at the top end of the distribution; however, men are also overrepresented among the lowest performers (Halpern, 2000).

Mathematics and science achievement is influenced not only by skills, but also by mathematics and science attitudes, test anxiety, and self-efficacy (Ashcraft and Moore, 2009; Geary et al., 2019). These dimensions are believed to be strong determinants of STEM careers and contribute to the underrepresentation of women in these fields (Moakler and Kim, 2014; Sax et al., 2015). Research has shown that men generally report more enjoyment and positive attitudes than women when engaging in mathematical activities (Ganley and Vasilyeva, 2011; Devine et al., 2012).

By contrast, women perform substantially better than men on verbal tasks (Moè et al., 2021), with girls using a broader vocabulary than boys on average by age two (Halpern, 2000; van der Slik et al., 2015). Verbal abilities comprise various skills, and gender differences are most prominent in the reading dimension, where the girls’ advantage is three times wider than the boys’ advantage in mathematics (Stoet and Geary, 2013). Nevertheless, Hirnstein et al. (2022) have cast some doubts on the magnitude of gender differences in verbal abilities claiming that publication bias might have influenced the results.

Cognitive abilities are largely interrelated. For example, high math skills predict higher reading scores and vice versa (Bos et al., 2012; Reilly, 2012). Women’s mean overall scores considerably outperform men’s, even though the latter appears to be better positioned at the top and lower tails of the distribution, a finding that supports the higher men variability hypothesis (Halpern et al., 2007; Bergold et al., 2017).

Turning to personality, gender differences are reported across the Big Five traits (openness, conscientiousness, extraversion, agreeableness, and neuroticism) and the HEXACO model (honesty–humility, emotionality, extraversion, agreeableness, conscientiousness, and openness), suggesting small to moderate gaps depending on the test and dimension analyzed. Specifically, women score higher in both neuroticism and agreeableness (Costa et al., 2001; Schmitt et al., 2008; Murphy et al., 2021), although findings have been inconclusive for openness, extraversion, and conscientiousness, with some studies showing women and others showing a men’s advantage (Goodwin and Gotlib, 2004; Shokri et al., 2007). The HEXACO model displays a similar pattern, with emotionality and honesty–humility both substantially higher in women than men (Lee and Ashton, 2004, 2018).

Men and women also differ in value priority and vocational interests. According to Schwartz’s theory (Schwartz, 1999), values define the motivations behind behaviors that regulate attraction in diverse fields. Although the variations are small to medium, research has consistently shown gender gaps, with men scoring higher in power, stimulation, hedonism, achievement, and self-direction and women scoring higher in universalism and benevolence (Schwartz and Rubel, 2005). On the other hand, vocational interests (Holland, 1997) describe how personality interacts with career environments and are important determinants of gender-typed career trajectories (Kuhn and Wolter, 2022). Previous studies have shown that men prefer to be employed in realistic fields, while women favor working with people (Lippa, 2010a), suggesting that men have more realistic and investigative interests, preferring careers in engineering, science, and mathematics. By contrast, women prefer “working with people” as they have more artistic, social, and conventional tendencies, which facilitate social science careers (Su et al., 2009).

### 3.2. Theories predicting that gender equality is linked with smaller gender differences

The *social role theory* (Eagly and Wood, 1999) posits that variations between men and women derive from the interaction, reinforced by socio-psychological processes, between evolved gender differences in physicality and the socio-cultural context in which these differences are expressed. Eagly and Wood (2012) have argued that, historically, men’s greater strength, endurance, and speed allowed them to conduct physically challenging duties. Conversely, women developed the ability to breastfeed, making them better suited for nurturing tasks. These evolved physical predispositions for specific activities shaped the domestic division of labor between men and women in ancient hunter-gatherer societies (Eagly and Wood, 2012).

As societies developed, the division of labor began to be influenced by physical gender differences in interaction with the social environment (Eagly and Wood, 1999). In modern countries, the socioeconomic setting dictates the relevance of those activities for which men and women have evolved peculiar physical predispositions. In this context, division of labor no longer relates solely to the domestic sphere but also encompasses paid labor, with men and women being segregated into different occupations. This gender segregation “derives in part from male and female biology—that is, mainly their evolved physical attributes, especially women’s reproductive activities and men’s size and strength, which can allow some activities to be more efficiently performed by one sex or the other depending on the socioeconomic and ecological context” (Wood and Eagly, 2013). Thus, the interaction of evolved physical gender differences with the social environment in which they are expressed is likely to be the main process shaping gender segregation.

Within societies, social-psychological processes reinforce gender segregation and make it appear “natural and sensible” (Wood and Eagly, 2013). Most people, when observing differential behaviors, assume that men and women are intrinsically dissimilar and construct specific “multifaceted” gender roles that include either essentially masculine or essentially feminine features (Beckwith, 2005; Wood and Eagly, 2012). Individuals then internalize these roles through societal mechanisms that reward people who comply and penalize those who deviate, leading both men and women to develop specific skills and personality (Friedman and Downey, 2002; Eagly and Wood, 2012). Consequently, gender differences in basic skills and personality are derived from the great effort that societies have undertaken to perpetuate gender segregation and comply with constructed gender roles (Wood and Eagly, 2013). It follows that in countries where gender roles are relaxed, gender segregation and, as a result, gender differences in basic skills and personality will be smaller (Eagly and Mitchell, 2004).

The *gender stratification hypothesis* (Baker and Jones, 1993) is consistent with the theory presented above. Although originally formulated to explain gender gaps in mathematics, it has also been applied in other spheres. The theory suggests that essentialist gender beliefs interact with individual goals, thereby generating gender differences. These differences emerge because men in patriarchal societies can connect their skills with career outcomes, whereas women cannot do so due to unequal opportunities (Else-Quest et al., 2010). In sum, societies that exhibit more gender stratification offer fewer opportunities for women to experience and develop the same skills and personalities as men.

Drawing from *expectancy-value theory* (Wigfield, 1994) and *cognitive social learning theory* (Bussey and Bandura, 1999), the gender stratification hypothesis argues that people undertake a task only if they value it and expect success. Perceptions of a task’s value are shaped by socio-cultural stereotypes about characteristics assumed to be gender-essential. Thus, women, due to gender stereotypes, would not find it valuable to invest in domains perceived as “masculine” because they would not expect to succeed in them. Instead, they would prefer to develop more “feminine” skills, and this predilection generates gender variances (Frome and Eccles, 1998).

The above process is ostensibly reinforced by environmental processes that highlight those behaviors that are generally linked to gender in a given cultural setting. In this context, environment relates to the social influences that could be imposed, selected, or contracted according to “levels of personal agency,” that is, the extent to which people feel they are in charge of their decisions (Bandura and Walters, 1977). According to this perspective, the immediate environment provides gender-essentialist information through parents, friends, and the media. Individuals regulate their behaviors according to the social expectations conveyed by this information and, through “direct tuition,” inform others about how different behaviors are linked to gender (Bussey and Bandura, 1999).

**Figure 1:**
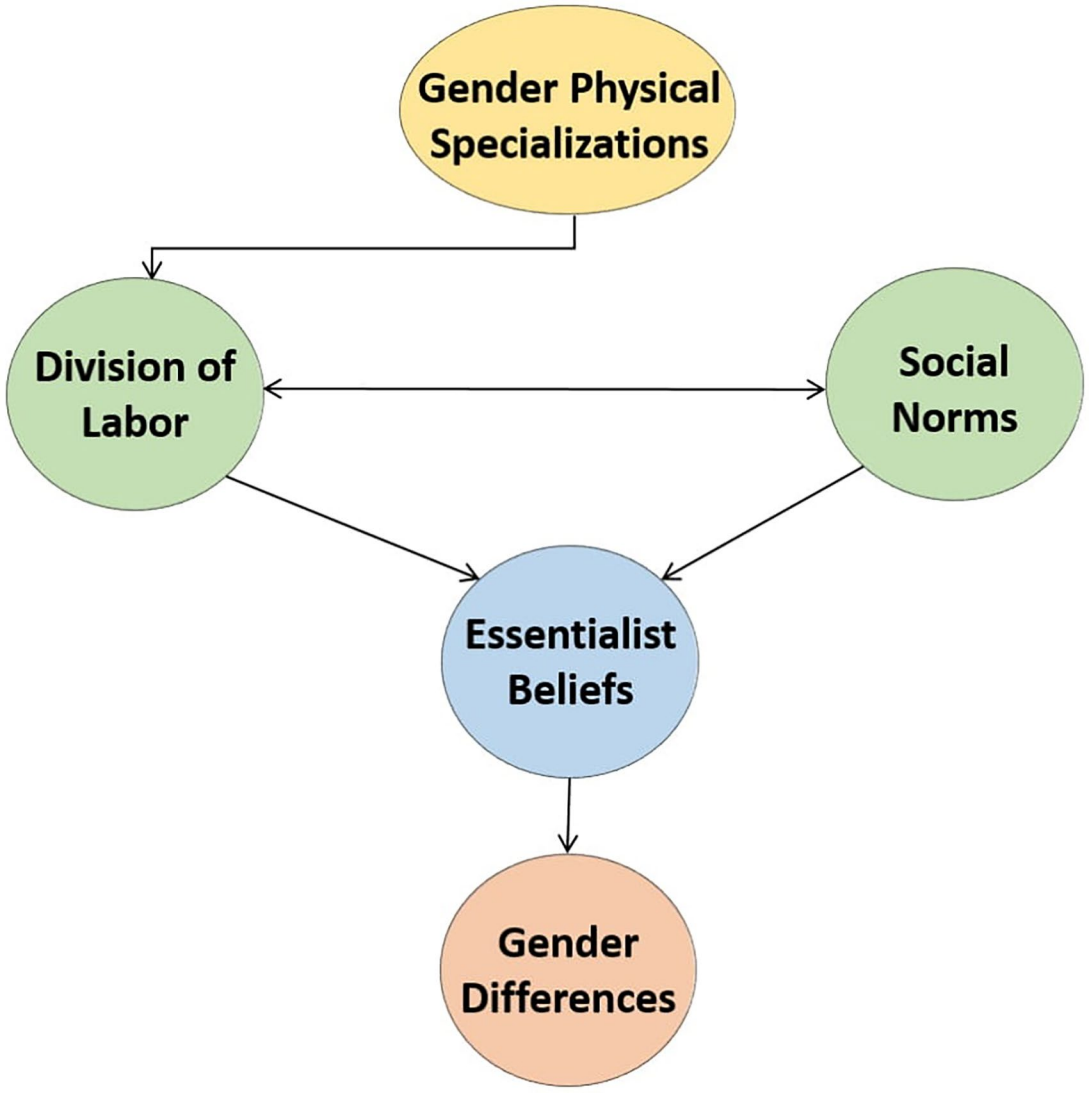
Overview of social-role theories of gender differences. Gender differences are generated by essentialist beliefs that men and women are intrinsically different which are in turn influenced by social norms in tandem with the division of labor derived from gender physical specialization.

According to the above theories, gender differences derive from false essentialist beliefs that diminish opportunities for subjective growth, making differences the result of unequal social treatment (Figure 1). Gender essentialism is conceived as a “powerful ideological” force that legitimates gendered choices and limits personal development (West and Zimmerman, 1987). Stated otherwise, gender not only represents the lens through which people see the world, but it also constitutes the basis for categorizing individuals (Bussey and Bandura, 1999). However, as the above theories emphasize, any visible variation between men and women results not from innate biological differences but from social impositions. If men and women were treated alike, gender stereotypes would fade, exposing them to similar stimuli and, consequently, eliminating gender differences in both basic skills and personality (Baker and Jones, 1993; Eagly and Wood, 1999). Thus, gender equality is likely to be associated with reduced gender variation. As Else-Quest et al. (2010) claimed, “where there is greater gender equity, gender similarities … will be evident.” Eagly et al. (2004) argued in the same vein, maintaining that “the demise of many sex differences with increasing gender equality is a prediction of social role theory.”

### 3.3. Theories predicting that gender equality is linked with wider gender differences

Drawing on gender essentialism, Charles and Bradley (2009) theorized an opposite effect—that gaps might increase with greater gender equality. They posited that, even if societies are gender equal, gender stereotypes endure because of the emphasis on individualism and self-expression in these societies. Specifically, gender equality stresses the expression of subjective preferences; however, it does not question how that preference emerges—an emergence that, Charles and Bradley (2009) ascribe to societal mechanisms influencing individuals based on their gender. These mechanisms strengthen essentialist beliefs about differences between men and women, in turn reinforcing gender-related roles (Levanon and Grusky, 2018).

According to the foregoing analysis, societal systems are characterized by internal structural diversification that is conceptualized to accommodate individual “expressive choices” but functions, instead, to increase stereotypes as people act out their internalized gender identities rather than their subjective preferences (Rawlings, 2007; Charles et al., 2014). In addition, long periods of care leave and advanced family policies, which are generally found in gender-equal countries, tend to influence horizontal gender segregation and compel women to enter into roles typically considered more gender-appropriate (Freiberg, 2019), widening even further the prevailing gender gaps. Thus, even when a society becomes more gender equal, “a preponderance of gender-typical choices” and an increase in gender variances can be expected (Charles and Bradley, 2009). Supporting this statement, some scholars have argued that gender stereotypes increase in more gender-equal nations (Breda et al., 2020; Napp and Breda, 2022). Others have stated that “cultural individualism” is often the strongest predictor of gender gaps in equal societies (Bleidorn et al., 2016; Kaiser, 2019).

Evolutionary theorists claim that differences between men and women are magnified in more gender-equal environments because privileged access to resources allows them to freely express specific gender “ambitions and desires” (Schmitt et al., 2008; Stoet and Geary, 2018). These theorists argue that from an evolutionary perspective, the possibility that men and women evolved with identical characteristics is a “theoretical impossibility” and maintain that gender differences are derived, in part, from innate predispositions (Vandermassen, 2011). Specifically, variations are expected to be visible in those domains in which the evolutionary pressure, mainly sexual selection, has influenced men and women differently (Schmitt, 2015). According to this view, the interplay between “sex-linked” genes and environmental stressors is responsible for the more pronounced gender dimorphism in modern nations (Schmitt et al., 2008).

In ancient hunter-gatherer societies, men and women evolved specific, intrinsic differences as a result of evolutionary adaptation (Mealey, 2000). Nevertheless, environmental conditions suppressed these innate differences that have subsequently re-emerged in developed societies characterized by reduced ecological pressure stemming from favorable economic circumstances. Gender differences in sensitivity to environmental change have played a key role in explaining this re-emergence. Generally, in the animal kingdom, the larger animal between the two sexes shows sharper fluctuations in behavior when ecological settings vary. The same appears to be true among humans, where men are more influenced by environmental changes (Teder and Tammaru, 2005). It follows that both men and women, but especially men, are less affected by environmental components in resource-rich countries, where they are free to follow their intrinsic characteristics (Schmitt et al., 2017). Conversely, in countries that offer fewer economic opportunities, choices are constrained, and reduced gender differences might be evident (Stoet and Geary, 2018).

Thus, according to the evolutionary hypothesis, increased gender variations in more gender-equal societies are mainly a product of the sexual selection that men and women have undergone during evolution together with gender differences in sensitivity to environmental changes (Schmitt et al., 2008). This interplay of gender-linked genes and environmental influences is relevant for some gender variances, such as height, since men in more developed societies are reported to be more sensitive to environmental changes (Sohn, 2015).

## 4. Basic skills and gender equality

Most studies on gender differences in basic skills have focused on the Trends in International Mathematics and Science Study (TIMMS) and the Program for International Student Assessment (PISA). TIMMS targets fourth- and eighth-grade students worldwide and reports their academic achievements every 4 years. Similarly, PISA is a triennial test of mathematics and science administered to 15-year-old adolescents in several countries. The PISA and TIMMS tests have been related to only a few gender equality indices; the most commonly used are the World Economic Forum’s Gender Gap Index (GGI) and the United Nations’ Gender Empowerment Measure (GEM). Both indicators are based on sub-indices that assess gender equality in numerous domains, such as educational attainment, political empowerment, and health.

### 4.1. Mathematics

As Table 1 shows, the math gender gap does not usually relate to gender equality when analyzing TIMMS data; in the PISA data, however, the findings appear to be more divergent.

**Table 1:** Correlations between mathematics gender differences (men > women) and both composite indices and specific indicators of gender equality.

References	Test	Year	Countries	Indices	Correlation
*Composite indices*					
Else-Quest et al. (2010)	TIMMS, PISA	2003	46, 41	GEM, GEQ, SIGE, GGI	Not Found/Negative
Fryer and Levitt (2010)	TIMMS, PISA	2003	47, 41	GGI	Not found/Negative
Kane and Mertz (2012)	TIMMS, PISA	2007 and 2009	52, 65	GGI, GEI	Positive
Hyde and Mertz (2009)	PISA	2003	30	GGI	Negative
Reilly (2012)	PISA	2009	34	GGI, RSW	Not found
Stoet and Geary (2013)	PISA	From 2000 to 2009	75	GEM, GGI	Not reliable
Stoet and Geary (2015)	PISA	From 2000 to 2010	41 to 74	GEM, GGI	Not reliable
Ireson (2017)	PISA	2012	65	GGI	Not found
Gevrek et al. (2018)	PISA	2012	56	GGI	Negative
Anghel et al. (2019)	PISA	From 2003 to 2015	73	GGI	Not found
Gevrek et al. (2020)	PISA	2012	56	GGI	Not found
Ghasemi et al. (2019)	TIMMS	2015	48	GGI	Not found
Reilly et al. (2019)	TIMMS	2011	45	GGI	Not found
*Specific indicators*					
Else-Quest et al. (2010)	TIMMS, PISA	2003	46, 41	RE, WR, WPEA, FPS, HMP	Not found Negative
Reilly (2012)	PISA	2009	34	WR	Negative
Penner and Cadwallader Olsker (2012)	TIMMS	1995	22	WE, WL	Negative Positive
Stoet and Geary (2015)	PISA	From 2000 to 2010	41 to 74	WR, WPEA, FPS	Negative Positive
Gevrek et al. (2020)	PISA	2006, 2012	56	RE, WR, WPEA, FPS, HMP	Negative Not found

GEM, Gender Empowerment Measure; GEI, Gender Equality Index; GGI, Gender Gap Index; GEQ, Gender Equality and Quality of Life; SIGE, Standardized Index of Gender Equality; RSW, relative status of women; RE, ratio of men to women in education; WR, women in research; WPEA, women’s participation in economic activities; FPS, female parliamentary seats; HMP, female’s higher labor market positions; WE, women’s parity in education; WL, women’s labor market participation.

Else-Quest et al. (2010) found that higher gender equality leads to slightly smaller differences between men and women in mathematics, although with variation across indices (*r* = 0.09–0.14). Similarly, Hyde and Mertz (2009) showed that more equitable index scores result in more women being among the top performers; however, their analysis used a small country sample and excluded Scandinavian nations (more on this below). Moreover, Gevrek et al. (2018) argued that moving toward gender equality predicts a reduced gender gap in mathematics in the part that cannot be explained by “observable characteristics,” that is, explained by elements that can be controlled for in statistical analyses.

However, the results appear to depend on the years that were considered in the analysis. For example, Stoet and Geary (2013, 2015) found that only the 2003 PISA assessment was consistent with theories hypothesizing that gender equality is linked with smaller gender differences. For other years, gender-equal practices were unrelated to a mathematics gap. Additionally, the results are sensitive to the inclusion of Scandinavian and gender-segregated, Muslim countries as well as gender-equal nations in which boys considerably underperform girls (Fryer and Levitt, 2010; Kane and Mertz, 2012; Stoet and Geary, 2015). However, some have raised doubts about including Muslim countries in the sample (Kane and Mertz, 2012). Other scholars have proposed that the positive findings derive from a spurious correlation between the GGI and country-specific unobserved variances (Anghel et al., 2019). Finally, as reported in Table 1, Gevrek et al. (2020) recently reversed their findings, strengthening the evidence that gender equality, measured by composite indicators, is not linked to gender differences in mathematics achievement.

However, composite indices may fail to account for explicit factors influencing the mathematics gender gap while specific indicators may be more suitable for measuring how gender differences vary in relation to gender equality. As Table 1 shows, having more women in research, higher levels of female participation in economic activities, a higher ratio of women to men holding parliamentary seats, and greater educational equality seem to predict reduced gender variation (Else-Quest et al., 2010; Penner and Cadwallader Olsker, 2012). More recently, Gevrek et al. (2020) extended their research by decomposing the mathematics gender gap into that which could be explained by “observable characteristics” and that which could not. Their finding suggests that the men-to-women ratio in tertiary education and the lower gender wage gap are not related to the explainable part of the gender gap, although they predicted a reduction in the unexplained part.

As mentioned earlier, also the findings for specific indicators depend on the year and countries considered. For instance, the results for the “women in research” indicator are unreliable because they sharply fluctuate across PISA assessments (*r* = −0.16, *r* = −0.68; Reilly, 2012; Stoet and Geary, 2015). The relation is mainly driven by countries that are, on average, less gender-equal but display lower gender discrepancies, such as Latvia, Serbia, Tunisia, and Thailand, as well as non-OECD nations (Reilly, 2012; Stoet and Geary, 2015).

Regarding “women’s economic activity,” Stoet and Geary (2015) analyzed four PISA assessments (2000, 2003, 2006, and 2009) and concluded that only the 2000 and 2003 results were consistent with theories predicting that gender equality is linked to smaller gender differences. In addition, “females in parliamentary seats” never reached statistical significance; only in the 2003 assessment did a link appear by excluding either non-OECD or Nordic countries from the sample (Stoet and Geary, 2015). Further, while Penner and Cadwallader Olsker (2012) showed that countries with more women participation in the labor force tended to have higher mathematics gender differences, the gender gap was not linked to gender equality in their analysis, contrary to the predictions. In sum, only “women in research” demonstrated a significant negative relationship with the gender gap in mathematics, although the magnitude of this relationship is in doubt. Additionally, the gender equality paradox had no empirical support when analyzing mathematics abilities. Girls outperformed boys in diverse socio-cultural environments, such as Finland and Qatar, demonstrating that egalitarian attitudes do not explain gender discrepancies in this dimension (Stoet and Geary, 2015). However, more gender equality had a positive effect on individuals, with both men and women increasing their mathematics scores in this context, without any specific advantages for either group (Kane and Mertz, 2012).

### 4.2. Mathematics attitudes and anxiety

In line with the gender equality paradox, mathematics attitudes and anxiety gender gaps are higher in gender-equal countries (Else-Quest et al., 2010; Stoet et al., 2016). Else-Quest et al. (2010) explained this phenomenon by arguing that mathematics anxiety is “a luxury, most often experienced by individuals who are not preoccupied with meeting more basic needs.” However, at the national level, both men and women tend to be less anxious about mathematics in equal societies, even though men benefit more from this lack of anxiety, enhancing gender differences as a consequence (Stoet et al., 2016). Only Goldman and Penner (2016) showed contrary results to that of the above research, arguing that gender differences in mathematics attitudes remain stable, even in gender-equal countries. Recently, Marsh et al. (2021) proposed that the gender equality paradox in these dimensions is “illusory” as it vanishes when accounting for country-level academic achievements and socioeconomic status; however, further studies are needed to support their argument. According to the women’s political representation index, gender-equal nations also have wider self-efficacy and motivation gaps. By contrast, other specific indicators, such as “equality in wages” and “parity in secondary and tertiary education,” predict smaller gaps (Else-Quest et al., 2010; Gevrek et al., 2020). Similarly, anxiety differences decline when there is equal political representation between men and women because women gain more than men in politically equal environments (Else-Quest et al., 2010; Gevrek et al., 2020).

In conclusion, gender equality is negatively related to gender differences in mathematics attitudes when analyzing composite indices; however, specific indicators are either inversely or directly related. It appears that pursuing equal political representation counteracts the results achieved by parity in wages and education, putting the overall advantage into question. Moreover, although self-efficacy and motivational gender gaps increase as equality is achieved in political representation, parity in tertiary education and wages shows an opposite trend.

### 4.3. Science, reading, and overall scores

Table 2 shows the science gender gap’s mixed results for composite indicators. Analyzing the GGI, Reilly (2012) concluded that the gender gap in science achievement decreases as gender equality increases (*r* = 0.29); nevertheless, men are better represented among the top scorers. By contrast, Ireson (2017) failed to replicate any meaningful relationships. However, a recent meta-analysis reported that gender-equal societies are characterized by “a pattern of higher male achievement, while for nations with lower gender equality, we see a pattern of higher female achievement” (Reilly et al., 2019).

**Table 2:** Correlations between gender differences in science, reading, and overall scores (men–women) and both composite indices and specific indicators of gender equality.

References	Test	Year	Countries	Indices	Correlation
**Science**
*Composite indices*
Reilly (2012)	PISA	2009	34	GGI	Negative
Ireson (2017)	PISA	2012	65	GGI	Not found
Reilly et al. (2019)	TIMMS	2011	45	GGI	Positive
*Specific indicators*					
Reilly (2012)	PISA	2009	34	WR, RSW	Negative/Not found
**Reading**					
*Composite indices*					
Reilly (2012)	PISA	2009	34	GGI	Not found
Gevrek et al. (2020)	PISA	2006, 2012	56	GGI	Positive
*Specific indicators*					
Reilly (2012)	PISA	2009	34	WR, RSW	Positive
Gevrek et al. (2020)	PISA	2006, 2012	56	RE, WR, WPEA, FPS, MP	Positive
**Overall scores**					
*Composite indices*					
Stoet and Geary (2015)	PISA	From 2000 to 2010	41 to 74	GEM, GGI	Not found
Ireson (2017)	PISA	2012	65	GGI	Not found
Eriksson et al. (2020)	PISA, TIMMS	From 2003	74	GGI	Positive
Stoet and Geary (2020)	PISA	From 2009 to 2015	55 to 71	GGI	Positive
*Specific indicators*					
Bergold et al. (2017)	TIMMS PIRLS	2011	32	WAE, TSER, WPLM, WR	Positive/Negative

GEI, Gender Equality Index; GGI, Gender Gap Index; GEQ, Gender Equality and Quality of Life; RSW, relative status of women; WAE, women’s access to education; TSER, tertiary school enrollment rate, boys/girls; WPLM, women’s participation in the labor market; WR, women in research; RE, ratio of men to women in education; WPEA, women’s participation in economic activities; FPS, female parliamentary seats; HMP, women’s higher labor market positions.

As reported in Table 2, also the specific indicators provide mixed results. No connection with the science gender gap is established for the “relative status of women,” whereas “women in research” is linked with increased gender differences (*r* = −0.39; Reilly, 2012).

These studies were based on inter-group comparisons, which may not have been appropriate for analyzing the relationship in question given the small mean gender gap in science. However, analyzing intra-individual strengths could move the debate forward because these are strongly related to career choices (Wang and Degol, 2017). Studies have shown that men are more likely to have higher abilities in mathematics or science than in reading, generating a “math tilt,” whereas women generate a “verbal tilt,” with differences more visible at the distribution’s right tail (Wai et al., 2018). In other words, although the mean gender variation in science approaches zero, an increasing number of men as compared to women have their top skill in science as opposed to reading, whereas the opposite trend holds true for women (see below). Analyzing 67 nations, Stoet and Geary (2018) pointed out that gender variances in science (and mathematics) intra-individual strength are higher in favor of boys in gender-equal nations. This trend among men could facilitate their preference for scientific careers because they would have the highest likelihood of success and especially so in gender-equal environments (Dekhtyar et al., 2018).

Regarding attitudes, “almost everywhere” girls display a lower science self-concept than boys, even when their academic skills are equal to those of their male peers (Sikora and Pokropek, 2012). Supporting the gender equality paradox, research has noted that gender differences in science self-efficacy, science enjoyment, and interest tend to be larger in gender-equal nations (Stoet and Geary, 2018; Liou et al., 2022).

Table 2 shows that studies on reading differences, although few, have substantially converged, demonstrating an increased gender gap in favor of women when there is more equality between genders. Although no correlation is found for the GGI, gender equality results in higher women representation among top-performing students (Reilly, 2012). Notably, the GGI has recently been linked to an increased reading gender gap in advanced societies (Gevrek et al., 2020). Analyzing specific indicators, Reilly (2012) showed that “women in research” directly relates to gender differences in reading achievement, thus predicting progressively higher variations. Gevrek et al. (2020) reached similar conclusions, arguing that the reading gender gap is wider in favor of girls in countries where there is more gender equality in the labor market. Furthermore, studies on intra-individual strengths have also been consistent, showing that girls’ tilt in reading skills is larger than that of boys in gender-equal societies (Stoet and Geary, 2018).

Few studies have focused on gender differences at the aggregate skills level, and those that exist have shown mixed results (see Table 2). Similar to the results for mathematics ability, Stoet and Geary (2015) found a significant increase in aggregate skill differences between boys and girls in nations with higher gender equality (GGI), although only in the 2003 PISA assessment. However, excluding either Iceland or Finland from the sample significantly weakened the link, and it disappeared when considering other years (Stoet and Geary, 2015; Ireson, 2017). Recently, inspired by research on gender differences in gray and white matter, Stoet and Geary (2020) argued that the basic skills pattern should be considered as a whole to understand the full magnitude of gender variation. Assessing the overall pattern in mathematics, science, and reading performance, it appears that the gap is greater than previously measured, corresponding to a large statistical difference, and it widens in more gender-equal environments.

Some researchers have proposed that egalitarian values, have a “more pervasive influence” and might offer a better understanding of the topic (Eriksson et al., 2020). An examination of these values suggests that “one standard deviation higher in gender equal values is on average 5.2 points more beneficial for boys” (Eriksson et al., 2020). This observation holds true for the GGI.

Contrary to theories predicting that gender equality is linked with smaller gender differences, “male/female enrollment in tertiary education” is inversely related to gender differences in overall achievement in countries with gender-neutral enrollment rates that also have more men among the top performers (*r* = 0.19; Bergold et al., 2017). Conversely, “women’s labor market participation,” “women’s share of research positions,” and “the ratio of women to men with at least a secondary education” have medium-size negative correlations (from *r* = 0.33–0.42), which may account for 28.7% of the gender variation (Bergold et al., 2017).

In sum, few studies have examined the link between gender equality and gender differences in science, reading, and overall scores, making it difficult to draw any firm conclusions. The findings for science and overall scores are contradictory, while for reading, there is substantial agreement about there being a gender equality paradox favoring women. Furthermore, due to their interrelatedness, a communal pattern between these skills emerges when examining intra-individual strengths. This pattern is characterized by increasingly wider science/mathematics and reading tilts for boys and girls, respectively. The tilt for girls shows that when girls have a science or mathematics score similar to boys, they tend to have better grades in reading, a trend that is especially observed in gender-equal nations (Stoet and Geary, 2018). However, scholars have only recently begun to consider intra-individual strengths, which represent a great opportunity for future studies on gender segregation.

## 5. Personality and gender equality

### 5.1. The big five and the HEXACO model

Evidence supporting a paradox emerged as early as 2001 when Costa et al. (2001) concluded that men’s and women’s personalities differ more in gender-equal countries. Schmitt et al. (2008) replicated these findings across 55 nations, again suggesting a positive correlation between gender differences and gender equality. More recently, larger gender differences in agreeableness favoring women have been found in gender-equal nations (see Table 3), mainly because of lower agreeableness in men in these nations with gender being the strongest predictor of individual levels (Lippa, 2010b). Conversely, the gender gap in neuroticism (women > men) has not been found to be affected by gender equality, even though the UN’s gender development and empowerment index predicts a decrease in negative emotions in both men and women (Lippa, 2010b).

**Table 3:** Correlations between gender differences in personality (men–women) and composite indices of gender equality.

References	Model	Year	Countries	Indices	Correlation
*Big Five and HEXACO models*					
Lippa (2010b)	Big Five	2005	53	UN	Positive/Not found
Mac Giolla and Kajonius (2019)	Big Five	2000	22	GGI	Positive
Kaiser (2019)	Big Five	2001 and 2011	50	GGI	Positive
Lee and Ashton (2020)	HEXACO	From 2014 to 2018	48	GGI	Positive/Not found
*Basic human values*					
Schwartz and Rubel-Lifschitz (2009)		2002 and 2004	25	PCC, PA	Positive
Fors Connolly et al. (2020)		2016	32	GEI	Positive
*Vocational interests*					
Ott-Holland et al. (2013)		2013	20	GLOBE Study	Positive/Not found
Tao et al. (2022)		2021	42	GEI	Positive

GGI, Gender Gap Index; UN, UN’s Gender-Related Development Index; PCC, Population Crisis Committee Index of Gender Equality; PA, Prescott-Allen’s (2001) index of gender equity.

While these findings are illuminating, looking only at single dimensions may lead to counterintuitive results because personality is multifaceted (Vianello et al., 2013). Although the average gender gap for a given personality trait is small, the overall variance is conventionally regarded as large, implying a significant difference between men and women (Del Giudice, 2009). Based on the latter premise, Mac Giolla and Kajonius (2019) noted a strong relationship between gender personality differences and gender equality, with overall differences being broader in “gender-friendly” countries (*r* = 0.69). Other studies have supported these results, observing the same widening pattern (Kaiser, 2019). Similarly, the emotionality gap from the HEXACO model displays a direct relationship with the GGI (*r* = 0.56), with women having an increasingly higher level than men in more gender-equal countries. However, honesty–humility fails to display any association with gender equality (Lee and Ashton, 2020).

Further evidence for a gender equality paradox in personality emerges from the study by Falk and Hermle (2018) that, building upon the above personality models, related gender differences in economic preferences – positive reciprocity, patience, altruism, trust, risk-taking (higher in women), and negative reciprocity (higher in men) – to gender equality measures. They concluded that the differences are characterized by sharp increases in more gender-equal countries (*r* = 0.67).

### 5.2. Basic human values and vocational interests

Basic human values (see Table 3) of power, achievement and stimulation are generally considered more important for men, whereas benevolence and universalism are valued among women. Past research has found that these gender differences are broader when men and women are treated equally, even though both genders regard masculine values to be less significant (Schwartz and Rubel-Lifschitz, 2009). More recently, Fors Connolly et al. (2020) extended the research on human values by adding a temporal dimension. Their analysis replicated the results cross-nationally, although temporal examination displayed a convergence between men and women in benevolence (over time, Cohen’s *d* −15%), with universalism and stimulation gaps remaining constant (Fors Connolly et al., 2020). However, as the authors noted, this convergence resulted from factors not linked to gender equality, indicating that the correlation might be spurious and caused by confounding factors related to both gender equality and personality. This additional finding suggests that gender equality could not cause gender differences in values and that the gender equality paradox needs further exploration.

For vocational interests, few studies have examined how gender differences change with gender equality. Using the Brinkman Model of Interests, one study found that ‘gender differences in musical and persuasive interests decreased in countries with high gender egalitarianism; nevertheless, clerical and scientific interests were higher when gender egalitarianism was high’ (Ott-Holland et al., 2013). However, most differences did not show any variance. More recently Tao et al. (2022) offered a more comprehensive overview highlighting that across all dimensions of vocational interest analyzed, increased gender equality was associated with wider gender differences. As Table 3 shows, gender personality differences generally increase in gender-equal countries. This finding is consistent across models and it appears to be valid also for dimensions not analyzed in this review (see Discussion for a more in-depth analysis).

## 6. Discussion

The systematic narrative literature review investigated recent studies on gender differences in basic skills and personality to determine whether cross-national relationships can be found with gender equality. The goal was to assess whether theories predicting that gender equality is linked with smaller gender differences have empirical support or whether a gender equality paradox has emerged in recent years. The general trend considers gender equality as either being connected to an increase in gender variations or having no relation with them, with a gender equality paradox occurring for gender gaps in some cognitive domains (attitudes toward mathematics, mathematics self-efficacy, mathematics anxiety, and reading) and personality.

### 6.1. Summary of the review

Based on the foregoing literature review, it can be seen that research supporting reduced gender differences in more gender-equal countries is scarce and inconsistent. A negative correlation is generally detected when analyzing gender differences in mathematics skills utilizing PISA data, although the correlation is influenced by either the year considered in the study or the sample country (see below). Moreover, “women in research” is the only specific indicator consistently negatively linked to the mathematics gender gap, albeit with disagreement about the strength of the association. Lastly, no connection between gender differences in mathematics and gender equality indicators is found when analyzing the TIMMS assessment. However, many studies have focused solely on mean differences in mathematics abilities, which are small or non-existent. Only Bergold et al. (2017) and Hyde and Mertz (2009) assessed the right tail of the distribution, where gender differences are more pronounced. This lack of studies on top performers highlights a gap in the research that needs to be filled. Also important is analyzing intra-individual strengths when studying the mathematics gender gap, as Stoet and Geary (2018) have emphasized.

Research supporting a positive link between gender variances and gender equality measures appears to be more robust and consistent. The literature on mathematics attitudes and anxiety shows that composite indicators predict a widening gender gap as equality between men and women advances. In addition, scholars agree that gender equality is connected with a larger advantage for women in reading and evidence further shows that gender personality differences are larger in more gender-equal nations. Men and women are less alike, especially in personality traits and basic human values, in countries that have invested the most in gender equality. Further support for a gender equality paradox in personality also emerges when examining other personality domains not included in this review. For example, wider gender gaps in self-esteem and narcissism (higher in men) exist in more gender-equal nations where women have more reproductive control, more executive positions, and their education is either similar to or higher than that of men (Bleidorn et al., 2016; Jonason et al., 2020).

Specific indicators are either directly or inversely related to the mathematics gender gap, raising doubt about them being related to a general advantage (Table 4). In addition, findings on science and overall scores are uncertain, even though both science anxiety and science intra-individual strengths follow a trend opposite to that anticipated by theories predicting a link between gender equality and smaller gender differences. Interestingly, other skills, such as episodic memory and visuospatial ability, show the same widening tendency, strengthening the case for a possible paradox in this area (Lippa et al., 2010; Asperholm et al., 2019).

**Table 4:** Summary of the papers included in the review.

**Basic skills**			
*References*	*Skills*	*Indices*	*Coverage*
Anghel et al. (2019)	Maths	GGI	73 countries worldwide
Fryer and Levitt (2010)	Maths	GGI	47 countries worldwide, no Middle Eastern countries in PISA
Gevrek et al. (2018)	Maths	GGI	56 countries worldwide with a focus on OECD Countries
Ghasemi et al. (2019)	Maths	GGI	48 countries worldwide, low African representation
Hyde and Mertz (2009)	Maths	GGI	28 countries worldwide, no Scandinavian or African countries
Kane and Mertz (2012)	Maths	GGI, GEI	65 countries worldwide with a focus on OECD Countries
Penner and Cadwallader Olsker (2012)	Maths	WE, WL	22 OECD countries mostly European
Stoet and Geary (2013)	Maths	GEM, GGI	75 countries worldwide
Ireson (2017)	Maths, Science	GGI	65 countries worldwide with a focus on OECD Countries
Reilly et al. (2019)	Maths, Science	GGI	45 countries worldwide, low African representation
Bergold et al. (2017)	Overall	WAE, TSER, WPLM, WR	17 countries, only Europe
Eriksson et al. (2020)	Overall	GGI	67 countries worldwide with a focus on OECD Countries
Stoet and Geary (2020)	Overall	GGI	71 countries worldwide
Stoet and Geary (2015)	Maths, Overall	GEM, GGI, WR, WPEA, FPS	74 countries worldwide
Else-Quest et al. (2010)	Maths, Attitudes	GEM, GEQ, SIGE, GGI	47 countries worldwide, no Middle Eastern countries in PISA
Gevrek et al. (2020)	Maths, Reading, Attitudes	RE, WR, WPEA, FPS, HMP	56 countries worldwide with a focus on OECD Countries
Reilly (2012)	Maths, Science, Reading, Attitudes	GGI, RSW, WR	Worldwide with a focus on OECD Countries
Stoet and Geary (2018)	Maths, Science, Reading, Attitudes	GGI	67 countries worldwide with a focus on OECD Countries
Goldman and Penner (2016)	Attitudes	GEM	49 countries worldwide, only 1 African nation
Marsh et al. (2021)	Attitudes	GGI	68 countries worldwide with a focus on OECD Countries
Stoet et al. (2016)	Attitudes	GGI	68 countries worldwide with a focus on OECD Countries
Liou et al. (2022)	Attitudes	GGI	22 countries worldwide, no Scandinavian or African countries
**Personality**
*References*	*Model*	*Indices*	*Coverage*
Lippa (2010b)	Big Five	UN	53 countries, low South American and African representation
Mac Giolla and Kajonius (2019)	Big Five	GGI	22 countries, low representation across continents
Kaiser (2019)	Big Five	GGI	50 countries worldwide with a focus on OECD Countries
Lee and Ashton (2020)	HEXACO	GGI	48 countries worldwide, no African countries
Falk and Hermle (2018)	Economic preferences	GEI	76 countries worldwide
Schwartz and Rubel-Lifschitz (2009)	Basic human values	PCC, PA	68 countries worldwide
Fors Connolly et al. (2020)	Basic human values	GEI	32 countries, only European
Ott-Holland et al. (2013)	Vocational interests	GLOBAL Study	20 countries worldwide, low representation across continents
Tao et al. (2022)	Vocational interests	GEI	53 countries low African representation

GEM, Gender Empowerment Measure; GEI, Gender Equality Index; GGI, Gender Gap Index; GEQ, Gender Equality and Quality of Life; SIGE, Standardized Index of Gender Equality; RSW, relative status of women; RE, ratio of men to women in education; WR, women in research; WPEA, women’s participation in economic activities; FPS, female parliamentary seats; HMP, female’s higher labor market positions; WE, women’s parity in education; WL, women’s labor market participation; WAE, women’s access to education; TSER, tertiary school enrollment rate, boys/girls; WPLM, women’s participation in the labor market; HMP, women’s higher labor market positions; UN, UN’s Gender-Related Development Index; PCC, Population Crisis Committee Index of Gender Equality; PA, Prescott-Allen’s (2001) index of gender equity.

### 6.2. Implications of the gender equality paradox

Understanding the possible reasons for the increase in gender differences in countries that promote gender equality is important and relevant since these countries may be leading men and women toward gendered trajectories, a path that is already observable in higher education. Charles and Bradley (2009) noted that the most advanced societies demonstrate more pronounced gender segregation in education. Stoet and Geary (2018) also observed that more gender-equal nations (measured by the GGI) have the widest gender gap among STEM graduates. Supporting these results, research has shown that gender differences using “interest in math careers” as a predictor of future major subjects are greater in countries with higher gender equality, with both men and women being, on average, less interested in mathematics than those in other countries (Goldman and Penner, 2016; Charles, 2017; Breda et al., 2020). The same pattern is observed in the job market, where horizontal segregation is more pronounced in more gender-equal environments (Blackburn and Jarman, 2006; Wong and Charles, 2020). Several investigations have documented this phenomenon and concluded that “Scandinavian countries are notable for their exceptionally high degrees of segregation” despite their advancement in gender equality (Jarman et al., 2012). However, more recent findings have also detected desegregation patterns in more gender-equal nations (Hustad et al., 2020).

### 6.3. The gender equality paradox: Possible explanations

The question of why gender differences are sometimes higher in more gender-equal countries remains. Some have proposed that the paradox in mathematics anxiety and attitudes might originate from the better economic conditions needed for these emotions to emerge. In countries where women are highly oppressed, these are more concerned about meeting more basic needs. Conversely, where economic, political, and educational circumstances are more favorable for women, anxiety toward mathematics activities is more likely to emerge (Else-Quest et al., 2010). However, at the national level, both men and women are less anxious about mathematics in developed, gender-equal countries, indicating that alternative explanations are needed (Stoet et al., 2016). In fact, others have suggested that, in gender-equal nations, men and women set aside financial drives and follow more intrinsic career interests because of easier access to economic resources. Hence, women are less exposed than men to STEM activities, “giving them less opportunity to reduce their negative feelings about mathematics” (Stoet et al., 2016).

With respect to reading abilities, the paradox might result from the interaction of two factors: the interrelation between basic skills and Western societies’ strong efforts to equalize boys’ and girls’ mathematics performance that has instead, paradoxically, increased reading skills in girls. Notably, where mathematics gender differences are reduced, the reduction is mainly due to an improvement in women’s reading (Guiso et al., 2008). It follows that countries with smaller mathematics gender differences have the largest reading gaps (Stoet and Geary, 2013). As mathematics is promoted in girls, their reading skills appear to benefit. However, because boys’ disadvantage in reading is, on average, less of a concern among policymakers, gender variations in this dimension have widened.

Some researchers have explained the gender equality paradox in personality by arguing that only differences in self-reported domains are increased (Eagly and Wood, 2012). Here, the reference-group effect (Heine et al., 2002) might conceal variances in less gender-equal countries, where men and women compare themselves with others of their own gender (Guimond et al., 2007). If this explanation holds true, the gap in gender-equal nations would be a better estimate of personality differences between the genders because in these nations both women and men have a more accurate comparative term that includes the whole population rather than just a subset (Schmitt et al., 2017).

Another explanation may be that personality is strongly culturally influenced. According to this view, individualism and self-expressive values act in tandem with gender stereotypes, promoting gender variance as individuals act out their “gendered self” (Charles and Bradley, 2009; Breda et al., 2020). This explanation of the gender equality paradox corresponds to the findings in gender-equal nations that cultural mechanisms are at play accommodating women-typical roles, such as job flexibility and high parental care—roles that encourage women to embark on gendered paths and experience more communal traits (Levanon and Grusky, 2018). Thus, it should not be surprising that, in gender-equal countries, men and women appear to differ more than in non-gender-equal countries and that this difference is expanding as women-typical roles are becoming more prevalent. Rather than expressing intrinsic gender differences, in these nations, there is a reinforcement of gender essentialist beliefs, which constitute an artifact of social expectations about how men and women should comply with gender stereotypes (England, 2010).

While this argument is somewhat persuasive, research aiming at linking gender stereotypes with gender equality suffers from several theoretical and methodological limitations. Often scholars apply broad assumptions and rely on a limited, as well as unreliable, set of items to capture latent dimensions of implicit stereotypes hidden in survey data. For instance, in their recent article Napp and Breda (2022) used solely one item to grasp an alleged stereotype that girls lack talent by arguing that systematic gender difference in answering the question would highlight “the magnitude of the (internalized) stereotype associating talent with boys rather than girls.” In addition, several studies have argued that stereotypes about group features, when measured reliably, appear to be accurate (Jussim et al., 2015; Moè et al., 2021). Löckenhoff et al. (2014) observed that perceived gender differences in personality substantially match those found in self- and observer-rated personality tests. The authors concluded that gender stereotypes constitute “valid social judgments about the size and direction of sex differences” that are more relevant than socialization processes and ascribed cultural gender roles (Löckenhoff et al., 2014). This is not to say that culture plays no role in the emergence of gender differences, but that the social mechanisms amplifying gender variances—mechanisms that social-role theorists have identified—also capture intrinsic gender differences.

Evolutionary theorists propose a different explanation for the gender equality paradox. As they argue, some gender variations are sensitive to context-related fluctuations, demonstrating a gene–environment interplay. In societies in which conditions are favorable, gender-specific genes flourish due to a lower prevalence of diseases, lower ecological stressors, and lower starvation rates. Per this view, wider gender gaps in gender-equal nations most likely “reflect a more general biological trend toward greater dimorphism in resource-rich environments” (Schmitt et al., 2008). If this explanation holds true, then heritability estimates will be higher in developed societies than in less-advanced cultures. Some evidence in this direction has recently emerged (Selita and Kovas, 2019); however, the “WEIRD” gene problem—that nearly all twin studies have been conducted among Western, educated, industrialized, rich, and democratic societies—represents an obstacle for generalizing results and making inferences about cross-cultural heritability differences (Henrich et al., 2010).

### 6.4. A novel socio-cultural evolutionary account of the gender equality paradox in personality

**Figure 2:**
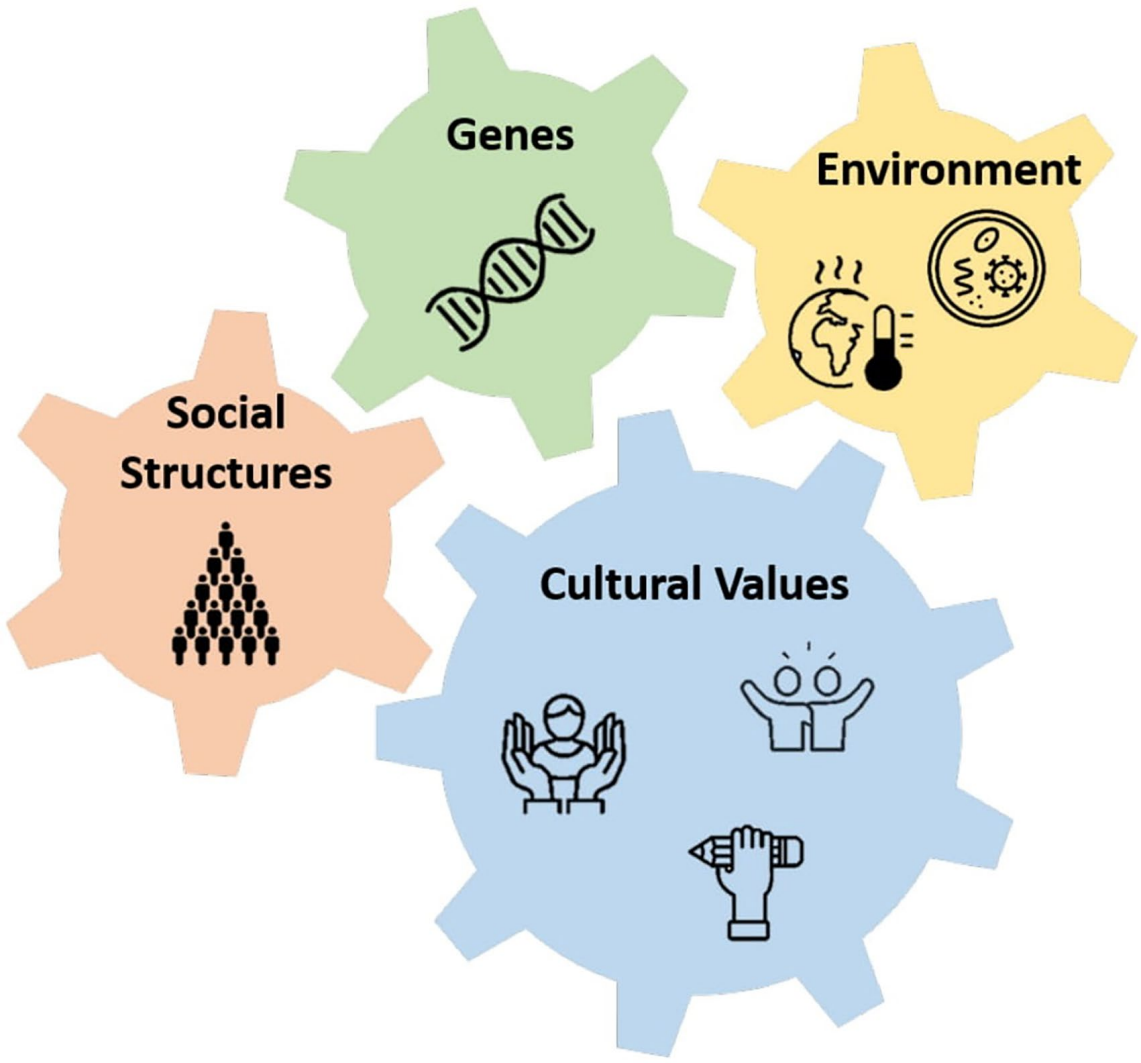
Socio-cultural evolutionary explanation of the gender equality paradox. The gears show the interrelations between gender-specific genes, social structures, and environmental components mediated by cultural values.

The present review proposes that the evolutionary explanation for the gender equality paradox might be more complex than it appears due to the presence of socio-cultural elements in the evolutionary process. As previously noted, genetic effects depend on the environmental conditions (diseases and ecological stress) under which they occur, yet the environment is embedded into society. Thus, the gene–environment interplay is enclosed within a cultural context with specific social norms and, by itself, cannot encompass all involved elements (Figure 2). Stated otherwise, the gene–environment interplay is a function of culture (Uchiyama et al., 2022). Therefore, gender-specific genes can be expected to be emphasized in societies embracing cultural values that would favor the expression of these genes. Consider, for example, individualism and self-expression. It is unsurprising that these values are related to the gender equality paradox, as Charles and Bradley (2009) have highlighted. In resource-rich environments that also value individualism and self-expression, intrinsic gender differences are more likely to emerge. This thesis points toward interpretation of Kaiser (2019), which states that both cultural individualism and pathogen levels confound the gender equality paradox in personality (see below). Also, Murphy et al. (2021) reached similar conclusions. A coherent, yet opposite, prediction might see gender differences as remaining stable or even decreasing in those resource-rich environments that culturally constrain self-expression. Accordingly, favorable cultural values would trump social mechanisms that amplify gender-based genes to emerge *via* a feedback-loop effect or “reciprocal causation” (Dickens and Flynn, 2001) according to which social structures adjust to distinct gender traits and vice versa, thus increasing gender differences.

### 6.5. Challenges for future cross-national research

While searching and analyzing the literature, this review also highlighted some challenges that researchers might face when conducting cross-national studies relating gender differences to gender equality measures. For mathematics ability, results could depend on outlier countries such as Scandinavian and gender-segregated, Muslim countries. In addition, the restricted country samples in international student assessments might be problematic. Despite the strong effort of PISA and TIMMS to be more inclusive, wealthy countries have traditionally been overrepresented, although the latest rounds have had very high coverage, including over 75 participating nations worldwide. Nevertheless, researchers, when assessing gender differences in mathematics abilities, should pay close attention to the countries included in their study because either the inclusion of outliers or a lack of heterogeneity might lead to biased estimations.

Another possible source of bias in research linking gender differences to gender equality on a cultural level is participant sample sizes, with some nations being overrepresented in comparison to others. How countries are clustered may also be problematic since countries are not independent data points and, “as such, they are like members of the same family or pupils of the same classroom” (Kuppens and Pollet, 2015). Therefore, appropriate statistical methods, multilevel modeling, for example, should be utilized to account for both unbalanced sample sizes and data structure.

Correlations between mathematics gender differences and gender equality might originate from a lack of country-level effects in the models. Anghel et al. (2019) argued that when time-invariant country unobserved heterogeneity is controlled for, no association between the two variables is found. Moreover, the link between gender equality and the gender gap in mathematics attitudes might be confounded by country-level academic achievements and socioeconomic status (Marsh et al., 2021).

Further, the gender equality paradox could be due to measurement error. Given that many international assessments and personality models have been developed in WEIRD countries, it is plausible that measurement error could be higher in non-WEIRD nations generating an illusory gender equality paradox. However, international assessments have been constructed to prevent such bias. For instance, PISA computes each student’s score based on a set of 5/10 plausible values designed to prevent measurement error and simplify secondary data analysis (Marsh et al., 2021). Also, the gender equality paradox in personality appears to hold even after correcting for measurement error (Kaiser, 2019; Fors Connolly et al., 2020; Tao et al., 2022). Nevertheless, when analyzing the link between gender differences in personality and gender equality, statistical procedures that control for measurement error should be applied (see for example Schmidt and Hunter, 2015).

Fors Connolly et al. (2020) highlighted the need for more temporal analyses of personality because an observed cross-national pattern may result from “a spurious relationship between gender equality and differences in personality” due to different country-level elements. Kaiser (2019) identified these elements as cultural individualism, food consumption, and historical pathogen prevalence levels. Other research has also agreed that cultural individualism could be a possible confounding factor as gender differences in personality are more pronounced in nations that highly regard individual self-expression (Costa et al., 2001; Schmitt et al., 2008; Tao et al., 2022).

Some scholars have called attention to the misuse of composite indicators of gender equality, raising several concerns thereof and arguing that they might not be suitable for empirical research (Else-Quest et al., 2010; Hyde, 2012). One concern is that these indicators, which encompass various domains from politics to economics, do not measure opportunities (Richardson et al., 2020). Another concern is that they are not interchangeable since they are differentially constructed. Thus, comparisons between research relying on different measures of gender equality might not be suitable. Some of the disparate findings concerning math ability might be driven by computational differences in the indices included in the analysis. Nevertheless, the gender equality composite indicators most commonly utilized (GGI, GEI, and GEM) show very high correlation coefficients (*r* ≥ 0.84), while other indicators substantially relate to one another, suggesting that, although some differences occur, these indices are similar in their ability to capture the general dimension of gender equality (Else-Quest et al., 2010; van Staveren, 2013; Stoet and Geary, 2015). Lastly, composite indicators may present a biased view of society due to the way gender equality is understood in the models. Often, disadvantages pertaining mostly to men are not taken into account when computing the indicators (Benatar, 2012). As an example of this bias, the GGI from the World Economic Forum assumes perfect gender equality in areas where women have an advantage over men. Specifically, values higher than 1, which would assume a men’s disadvantage, in each sub-index are capped. Thus, a more simplified approach to measuring national gender inequality is preferred (Stoet and Geary, 2019).

In addition, methodological issues also arise when using these indices. Some scholars have pointed out that correlations between gender gaps and the indices of gender equality could be driven by the strong economic component in these indices (Fors Connolly et al., 2020). Therefore, it is important to control for appropriate economic indicators, such as GDP *per capita* and the Human Development Index, when linking gender differences with gender equality (Kuppens and Pollet, 2015). Another difficulty may arise when contrasting results between composite indices and specific indicators occur. For mathematics attitudes, for instance, although composite indices suggest a gender equality paradox, specific indicators are either positively or negatively related to the gender gap. This may suggest that composite indices either capture an overall influence of gender equality or are unsuitable for evaluating gender differences. However, evaluation may lie outside the scope of models using these indices. Research linking gender differences with gender equality indicators has not tried to explain the paradox emerging from the analysis on the basis of gender equality *per se*; instead, it has just highlighted a paradoxical pattern that would otherwise have remained concealed. Since no theory has been put forward that fully unravels the paradox, further studies are needed.

Theories considered in this review that predict that gender equality is linked with smaller gender differences do not offer a valid explanation of gender differences in basic skills and personality. In addition, for some dimensions, the gender equality paradox raises further questions about how gender variation emerges, which calls for a new approach. Based on these premises, this review explored both social-role and evolutionary hypotheses and suggested new insights that combine these views, while also highlighting explanatory variables that might cause bias in the results. Thus, specific research that more closely examines the explanations proposed is needed, especially studies with an interdisciplinary focus. Notably, Fors Connolly et al. (2020) highlighted the importance of cross-temporal analyses of the gender equality paradox because these may reveal a different path. Since country comparisons may be insufficient for fully grasping the evolution of the paradox, future research should include a thorough cross-temporal examination for a more comprehensive understanding.

Lastly, the gender equality paradox is an emerging phenomenon that has gained substantial scientific support across subjects (Falk and Hermle, 2018; Campbell et al., 2021; Block et al., 2022; Vishkin, 2022). It requires attention from both the scientific community and the public because attempting to close gender gaps following traditional social-role theories and applying conventional methods, might end up exacerbating gender variations. In addition, the general pattern of increased gender differences in more gender-equal countries might inform that achieving equal opportunities does not go hand in hand with a reduction of gender gaps. Thus, policymakers should consider this trend when justifying interventions attempting to achieve equality of outcome between men and women.

## Data availability statement

The original contributions presented in the study are included in the article/supplementary material, further inquiries can be directed to the corresponding author.

## Author contributions

The author confirms being the sole contributor of this work and has approved it for publication.

## Funding

The work was supported by the Finnish National Board for Education through a working grant.

## Conflict of interest

The author declares that the research was conducted in the absence of any commercial or financial relationships that could be construed as a potential conflict of interest.

## Publisher’s note

All claims expressed in this article are solely those of the authors and do not necessarily represent those of their affiliated organizations, or those of the publisher, the editors and the reviewers. Any product that may be evaluated in this article, or claim that may be made by its manufacturer, is not guaranteed or endorsed by the publisher.
